# Supplementary figures and images for: Aging Shapes the Population-Mean and -Dispersion of Gene Expression in Human Brains
Source: Front Aging Neurosci. 2016 Aug 3;8:183. doi: 10.3389/fnagi.2016.00183 (PMC4971101; doi:10.3389/fnagi.2016.00183)

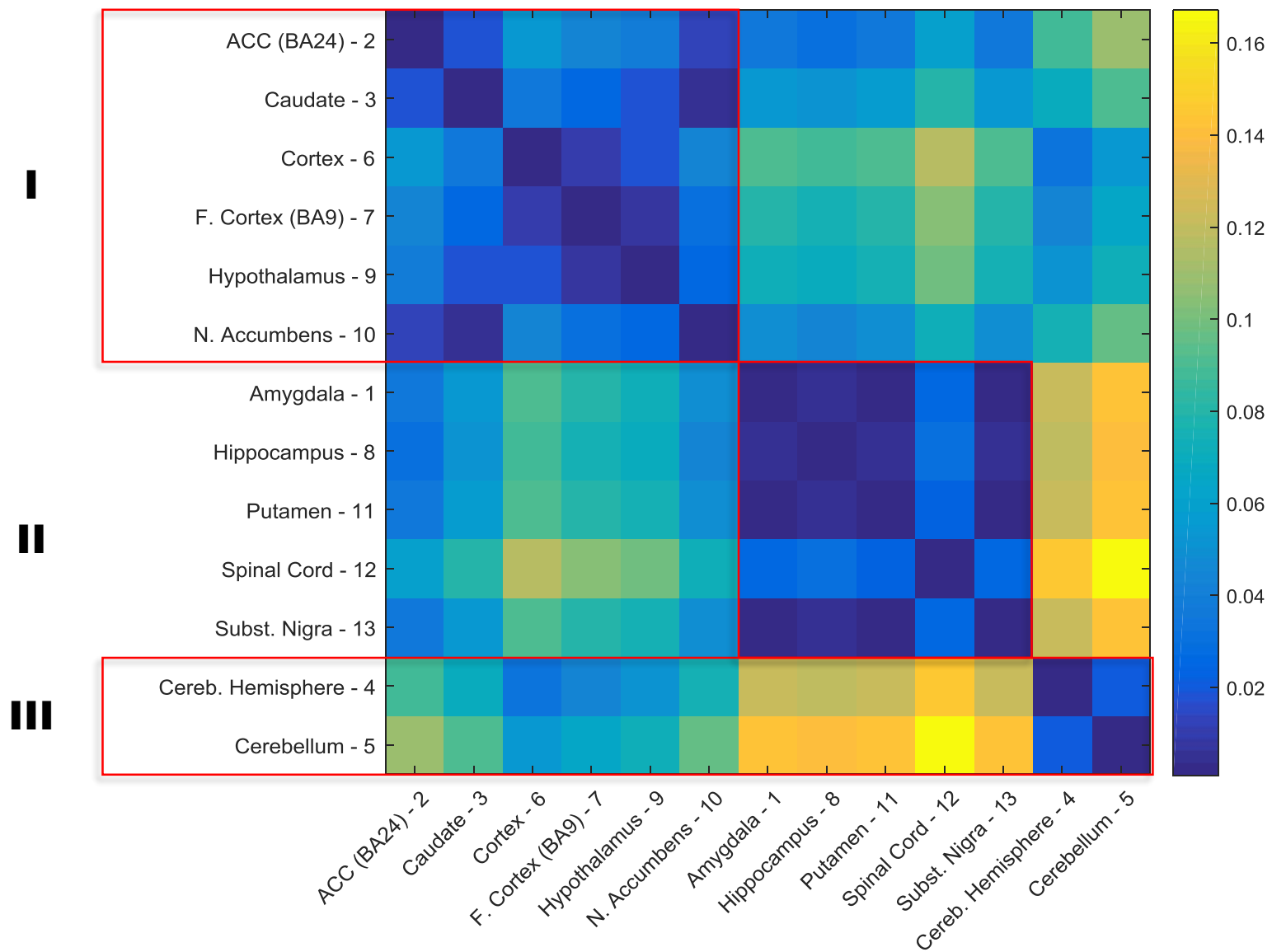

Supplement: Supplementary Figure S1 — Clustering based on the Euclidean distance between gene expression profiles. [file Image1.PDF]

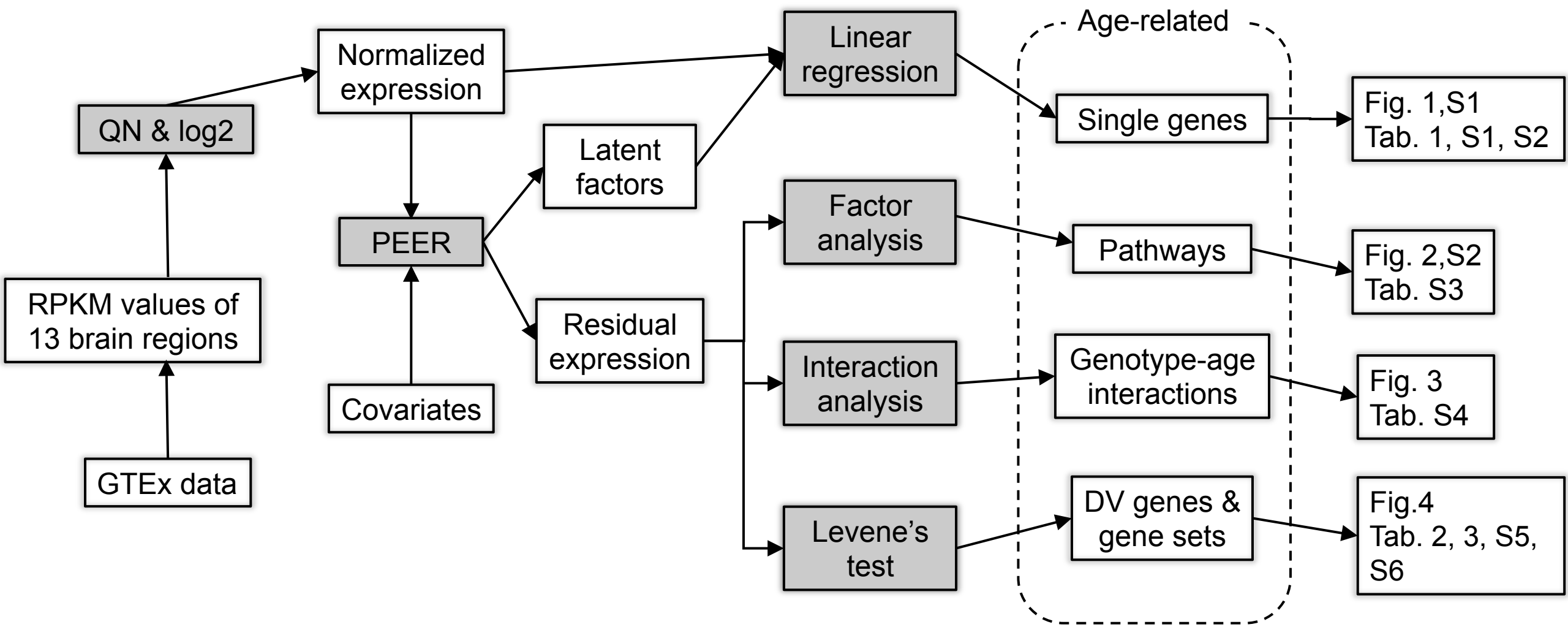

Supplement: Supplementary Figure S3 — Flowchart summary of methods described in this paper. Output can be found in the figures and tables indicated on the far right-hand side of the figure. [file Image3.PDF]

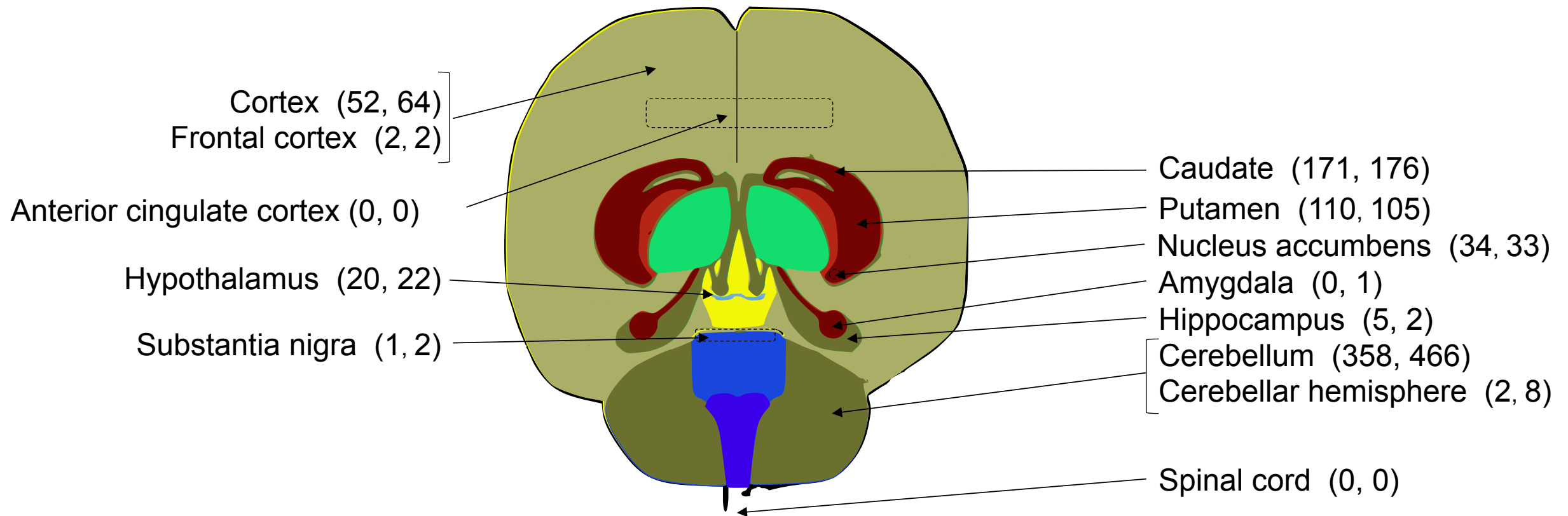

Numbers of age-related genes (up-regulated, down-regulated) at FDR 5%

Supplement: Supplementary Figure S4 — Diagram showing regions of the brain with age-related gene expression differences indicated in parentheses. Here, we show numbers of age-related genes that are up- or down-regulated at FDR 5%. Approximate locations of some regions are delineated with a dashed-line border, as not all regions are located within the same plane. [file Image4.PDF]
