# Supplementary material for: Aging Shapes the Population-Mean and -Dispersion of Gene Expression in Human Brains
Source: Front Aging Neurosci. 2016 Aug 3;8:183. doi: 10.3389/fnagi.2016.00183 (PMC4971101; doi:10.3389/fnagi.2016.00183)

REVIGO Gene Ontology treemap

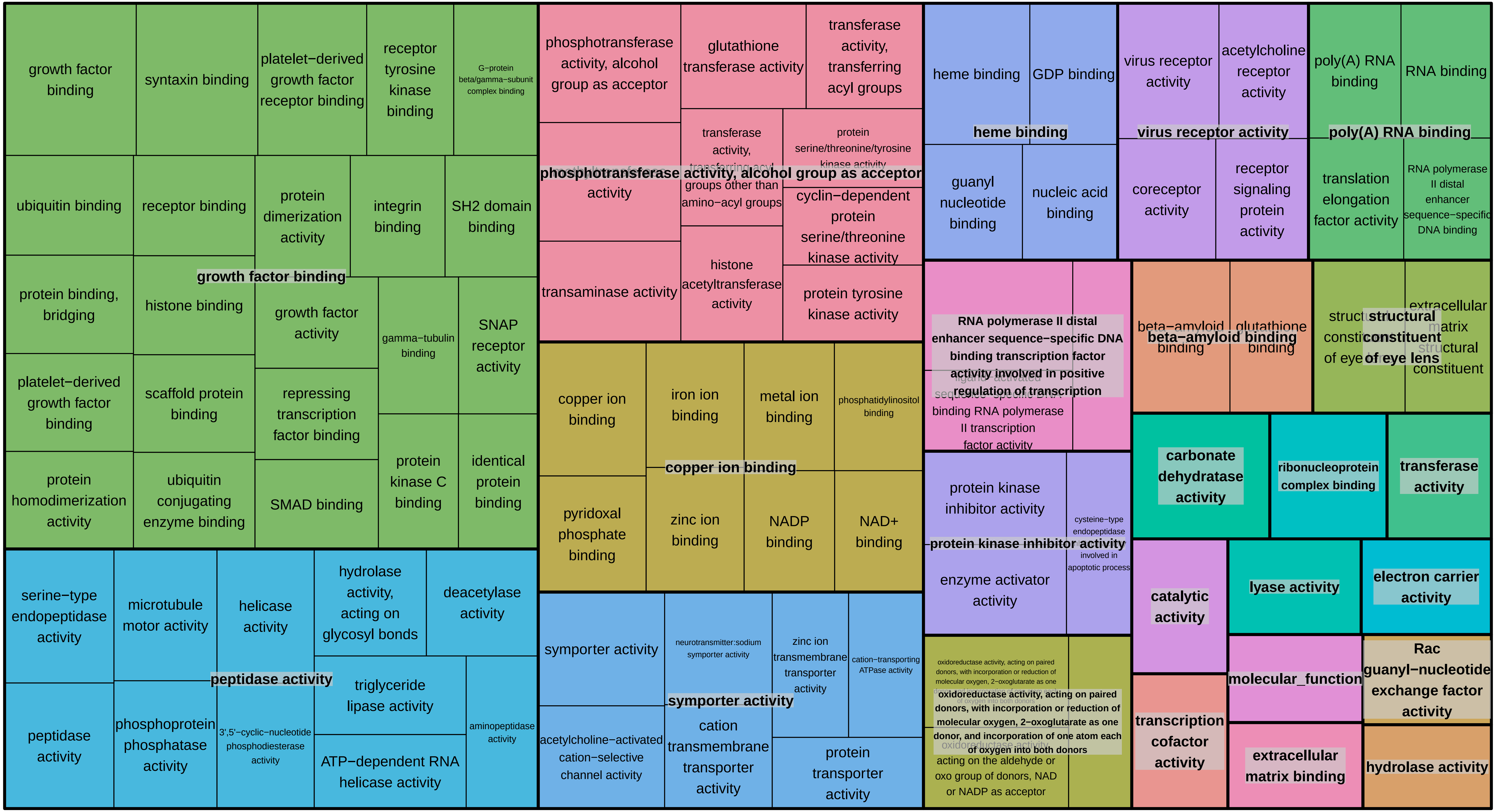

Supplement: Supplementary Figure S2 — TreeMap view of GO-term clusters generated by REVIGO, showing top-level molecular function GO terms of age-related gene sets at 5% FDR significance. [file Image2.PDF]
